# Exploring the Transformative Aftereffects of Religious Experiences on Pilgrims Along the Camino de Santiago in Spain

**DOI:** 10.1007/s10943-024-02127-z

**Published:** 2024-10-08

**Authors:** Snežana Brumec, Piotr Roszak

**Affiliations:** 1 Department of Sociology, Faculty of Arts, University of Maribor, Koroška cesta 160, 2000 Maribor, Slovenia; 2 Faculty of Teology, Nicolaus Copernicus University, Torun, Poland

**Keywords:** Camino de Santiago, Religious experience, Transformative aftereffects, Pilgrimage, Spirituality

## Abstract

This study examines religious experiences (REs) and their transformative aftereffects (TAs) among pilgrims on the Camino de Santiago. Analyzing 32 travelogue testimonies and survey data from 501 participants, it identifies three key dimensions of post-RE transformations: heightened Unity and Love, a strong commitment to the Apostolic Mission, and expanded Spirituality, Wisdom, and Detachment. Pilgrims report a shift toward greater love, unity with nature, and sharing insights, as well as increased spirituality and reduced materialism. The study finds significant correlations between TAs and individuals prioritizing their relationship with God and regular religious practices, highlighting REs’ impact on societal values and beliefs.

## Introduction

This article delves into the religious experiences (REs) and the transformative aftereffects (TAs) of pilgrims along the Camino de Santiago, designated as the inaugural European Cultural Itinerary by the Council of Europe in 1987. This pilgrimage route has witnessed a remarkable surge in pilgrim numbers, exceeding 446,000 by 2023, as reported by the Pilgrim’s Reception Office (2024). These figures encompass those who reached the city and obtained their Compostela certificate^1^ issued by the Cathedral of Santiago de Compostela. The burgeoning popularity of the Camino de Santiago has attracted considerable attention from the research community, resulting in numerous studies investigating various aspects of this pilgrimage.^2^ This surge in interest can be attributed to fundamental characteristics of late modern society, which significantly contribute to its widespread appeal (Brumec, 2024b). Therefore, the substantial increase in pilgrim numbers on the Camino de Santiago can be sociologically understood as a phenomenon rooted in broader social transformations, notably an increasing emphasis on experiential knowledge and a heightened significance of emotions. However, it is important to note that this increase is also influenced by a variety of other factors. Geographic aspects, historical developments, religious shifts, health trends, socio-economic progress, geopolitical changes, and advancements in technology and transportation all play crucial roles in shaping the growing popularity of the pilgrimage (e.g., Lois González & Lopez, 2021; Mróz, 2017; Santos, 2021). These multifaceted influences together contribute to the contemporary surge in Camino de Santiago pilgrimages.

Brumec et al. (2023) proposed that the concept of Exceptional Human Experiences (EHEs) could effectively capture and analyze the experiences of pilgrims and their enduring impacts. EHEs are understood as combinations of exceptional experiences (EEs) encountered during a pilgrimage and the transformative aftereffects (TAs) these experiences bring about. Their study involved qualitative and quantitative content analyses of 32 pilgrim travelogues, resulting in the development of an empirically grounded typology encompassing EEs, TAs, and their combinations (EHEs). They identified nine basic EE types: (1) Experience of interconnectedness, (2) Experience of deep calm and reconciliation, (3) Religious experience, (4) Here-and-now experience, (5) Experience of trust, (6) Cathartic experience, (7) Experience of meaningful coincidence, (8) Experience of insight, and (9) Experience of a dream vision. Additionally, they categorized eight core types of TAs: (1) Unity and Love, (2) Stronger interpersonal relationships, (3) Spirituality, Wisdom, and Detachment, (4) Vulnerability, (5) Apostolic mission, (6) Letting go, (7) Strengthened intuition, and (8) Boost of self-confidence.

Through their research, Brumec et al. (2023) established correlations between individual types within these two categories, substantiating the assertion that EEs during the pilgrimage serve as causal factors for TAs experienced after the pilgrimage. Furthermore, their findings concluded that all observed EEs are integral components of EHEs. This typology was later quantified by Lavrič et al. (2022) through an online survey involving 501 Camino de Santiago pilgrims, which highlighted the prevalence of EHEs among these pilgrims.

This article focuses on exploring the religious experiences and their transformative aftereffects among pilgrims of the Camino de Santiago. Utilizing insights from pilgrim-authored travelogues and bolstered by survey outcomes, the present study seeks to enrich comprehension of these phenomena. By elucidating their significance in facilitating emotional shifts marked by increased Unity and Love, shaping moral and cognitive frameworks, fostering heightened Spirituality, Wisdom, and Detachment, and inspiring pilgrims to share newfound insights and wisdom, our goal is to provide a nuanced understanding of specific dimensions of religious experiences.

## Theoretical Background

### Subjective and Emotional Turn

In the era of modernity, intellectualization and rationalization, as explained by Weber (2004), have reshaped our understanding of existence, leading to a belief in universal, comprehensible laws and a sense of disenchantment with the world. Concurrently, within late modern culture, marked by individualism and consumerism, a noticeable transformation termed *the subjective turn* is occurring. (Taylor, 2007).

This shift, observed by scholars like Shils (1961), emphasizes openness to sensation and sensibility, fostering what is termed *an experiential turn*. This societal change, epitomized by the depiction of our era as *an experience society* (Schulze, 1992), values personal experiences highly. Bauman (2000) notes that the prominence of subjective experience underscores the fluidity of modernity’s *liquid phase*, where experiencing things firsthand is central.

For Giddens (1991), subjective experiences shape one’s identity profoundly in late modernity. This shift toward subjective experiences is observed by Motak (2009) as a growing *obsession with experience* fueled by consumerism. Similarly, Heelas and Woodhead (2005) describe it as *a subjective turn in modern culture*, away from external roles toward lives influenced by inner motivations.

Yet, Weber (2004) views the pursuit of experience as indicative of a deficiency—a reluctance to embrace the mundane reality, following the disenchantment of traditional beliefs, fostering a prevailing ethos characterized by manufactured happiness. In addition to the subjective turn, late modernity witnesses *an emotional turn*, as described by González (2017). This resurgence of emotion, also known as the *affective turn* (Lemmings & Brooks, 2014), seeks to re-enchant a disenchanted world, valuing emotions once distrusted in a rational world.

Late modern spirituality, according to Motak (2009), emphasizes direct, emotive encounters over mere beliefs, prioritizing immediacy through emotional engagement. This emphasis on subjective experiences aligns with Simmel’s (1989) observation that spirituality is deeply intertwined with individual experience, tracing back to burgeoning individualism in early modernity. Luhmann (1998) notes that religion exists in social communication, where individual experiences merge into a collective understanding, although individualistic spirituality presents challenges in communication. This challenge is perhaps also evident in how religion is treated under the concept of cognitive safety (Roszak et al., 2024).

Ward (2004) suggests a shift from exclusive dogma to inclusive religiosity grounded in personal experience, celebrating multiplicity while asserting spiritual truth’s unity. Sheldrake (2007) posits that spirituality embodies the deepest values and meanings, reflecting the subjective shift toward individual self-actualization in modern Western society. Empirical evidence supports a widespread *spiritual turn* in Western countries (Houtman & Aupers, 2007, 2008), with subjective experiences being incorporated into spirituality (Knoblauch, 2006). At the same time, spirituality is sometimes contrasted with religion, although in classical theology it was understood as a form of living religion and not a substitute for it (Roszak & Mróz, 2024).

The emotional and subjective shift in late modern society influences modern spirituality. This shift is characterized by two tenets of late modern spirituality identified by Watts (2022). Firstly, the tenet of Immanence of God or the superempirical prioritizes emotional experiences over logical reasoning and doctrines. Research by Brumec (2024b) on Camino de Santiago pilgrims demonstrates the significant role of this tenet in their spiritual experiences, fostering a sense of interconnectedness and a direct connection to the divine.

Secondly, according to Watts (2022), late modern spirituality embraces experiential epistemology, valuing personal experiences as the primary source of knowledge. Pilgrims on the Camino de Santiago experience EHEs, embodying ecstatic and effervescent qualities such as self-transcendence and absolute joy. These findings align with the shift toward subjective experiences as a source of truth in late modern spirituality.

### Religious Experiences and Transformative Aftereffects

The exploration of religious experiences traverses a diverse landscape, encompassing emotional, cognitive, and transformative dimensions. Stark and Glock (1968) laid the foundation, defining religious experience as deeply affecting individuals emotionally. Building upon this emotional depth, Azari and Birnbacher (2004) emphasize the complexity of emotions in shaping religious experiences, rejecting reductionism, and proposing a deeper understanding rooted in feeling. Transitioning to cognitive processes, Mitchell (2017) delves into the intrinsic relationship between emotions and evaluative beliefs, setting the stage for Hoelter’s (2021) exploration of religious experiences as catalysts for personal transformation.

Hoelter’s insights draw from neurological research, advocating for a balance between intuitive and rational hemispheres of the brain (2021). His discussion, enriched by the perspectives of scientists like McNamara and Wildman, offers fresh interpretations of Rudolf Otto’s concept of mysterium tremendum, shedding light on the evolutionary origins of intense religious experiences. Watts (2020) extends this discussion, proposing a two-phase evolution of religion mirroring shifts in human cognition from intuitive to rational modes.

Hewitt (2012) brings attention to the emotional and biological underpinnings of religious experiences, advocating for a holistic approach that integrates altered states of consciousness. Nyaku’s (2022) offers a unique philosophical perspective, framing religious experiences as rooted in love and transcending the physical realm through intuition. Oberg (2022) further navigates the complexities of religious experiences, proposing the existence of a core self as the foundation for these encounters.

Tietjen (2017) explores mystical experiences as transformative journeys, challenging conventional understandings of reality and fostering unity with the universe. Vieten et al. (2006) highlight the transformative power of interconnectedness and oneness, amplifying compassionate tendencies and fostering altruism.

Scorgie et al. (2022) offer a real-world context, examining how spiritual experiences influence individuals’ decisions and perceptions within a religious community. Van Leeuwen and van Elk (2019) distinguish between general and personal religious beliefs, shedding light on the shaping factors of individual belief systems. Wilt et al. (2019) delve into sacred moments, unveiling core truths that structure perceptions and foster spiritual growth. Seitz (2024) argues that belief is a fundamental brain function, integrating environmental information with personal perspectives, thus emphasizing the cognitive underpinnings of religious beliefs.

Snow et al. (2023) explore divine grace as a mechanism of transformational growth, revealing its impact on humility and internal change. Emmons et al. (2017) underscore the psychological significance of divine grace, emphasizing its role in characterological changes and well-being. Gutierrez et al. (2017) conclude with a cross-faith comparison, highlighting the impact of religious and spiritual experiences on individuals’ narratives and lives. The transcultural nature of pilgrims’ experienced spiritual transformations is indicated by recent research among Chinese pilgrims to Compostela. According to this study, spirituality is associated with an increase in the intensity of their relationship to the transcendent, to others, to nature and to themselves, contributing to ecstatic perception of spirituality (Zhang et al., 2024). Collectively, these perspectives offer a nuanced comprehension of religious experiences and their transformative impacts, underscoring the imperative for additional inquiry into this increasingly relevant phenomenon in late modernity (Roszak, 2023).

## Present Study

The present study aims to bridge a significant gap in current research by examining the TAs of REs, specifically within the framework of the Camino de Santiago pilgrimage. With a primary focus on elucidating the REs encountered by pilgrims along the Camino de Santiago and their subsequent TAs, the research aims to address two core questions: Firstly, what types of REs do pilgrims undergo on the Camino de Santiago, and how frequently do they occur? Secondly, what are the TAs of these experiences, and how frequently do they manifest? By delving into these inquiries, the study contributes to advancing our understanding of religious experiences, pilgrimage practices, and their broader implications for personal growth, spiritual well-being, and cultural contexts.

### Study Methods

To achieve the objectives of this study, we draw on data from two key sources^3^ related to the Camino de Santiago pilgrimage: an analysis of 32 travelogue testimonies^4^ (Brumec et al., 2023) and survey responses from 501 pilgrims (Lavrič et al., 2022).

The first foundational research (Brumec et al., 2023), which forms the basis for this study, involved a comprehensive qualitative and quantitative content analysis of 32 travelogue testimonies documenting the Camino de Santiago pilgrimage. These travelogues, authored by pilgrims and published in Slovene up to the end of 2018, were accessed through the Slovenian library information system COBISS.

Among these testimonies, the majority (23) were authored by Slovenian writers, while six were written by authors from various countries and subsequently translated into Slovene. It is important to note that one book by a Slovenian author was in fact written by one of the authors of this article, who had firsthand experience as a pilgrim on Camino. This had an impact on the present analysis, for it added to both the validity of our interpretations and much-appreciated sincerity, which is a crucial component within qualitative research (Tracy, 2010, p. 7). The seven foreign authors include two French individuals (Potdevin, 2013; Rufin, 2016), three Americans (Gray & Skeesuck, 2017; MacLaine, 2000), one Croatian (Kapetanović, 2017), and one Irish author (McManus, 2014).

In their work, Brumec et al. (2023) developed a typology of EEs and TAs through an inductive approach facilitated by computer data processing. They employed the QDA Miner software for this purpose, importing and encoding the text data from the books in RTF files. Text coding formed the core of data processing. In the initial step, single-stage open coding was utilized to establish inductive, content-driven concepts by examining incidents or activities as potential indicators of the pilgrimage experience. A total of 346 codes were identified, firmly rooted in the data. In the subsequent step, these concepts were organized into categories that pertained to similar phenomena, resulting in more elevated and abstract codes. The final coding framework comprised a total of 29 categories. Various reported unusual experiences, such as interconnectedness, were categorized under EEs, while distinct effects, like the enhancement of intuition, were situated within TAs. These two categories were aligned with the theoretical framework of EHEs.

Subsequently, the relationships between these codes were further explored by calculating similarity indices and co-occurrence indices, enabling the execution of cluster analysis and multidimensional scaling on selected codes. This iterative process culminated in the development of a well-founded typology encompassing nine types of EEs, eight types of TAs, and their various combinations, collectively referred to as EHEs. One type of EE identified was religious experience.

The empirical analysis of this study centers on examining codes identified as REs and exploring the prevalent TAs generated by these experiences. To fortify our findings, we leverage pertinent quotations extracted from analyzed travelogues, which are incorporated into implementation matrices. These quotations provide an illustrative depiction of our research outcomes, complemented by relevant studies discussing these concepts.

Additionally, to gauge the frequency of REs during the pilgrimage and TAs in everyday life following the pilgrimage, we integrate data from the second foundational study (Lavrič et al., 2022). This study involved a survey of 501 participants, focusing on pilgrims who covered a minimum of 300 km along the Camino de Santiago pilgrimage route and represented various countries with proficiency in English. The participants were selected through purposive sampling from four English-speaking Camino de Santiago Facebook groups: American Pilgrims on the Camino (over 26,000 members), Australian Pilgrims on the Camino de Santiago and Beyond (over 4,300 members), Camino de Santiago All Routes (over 55,700 members), and Camino de Santiago (over 30,300 members). Data collection occurred from April to July 2020.

The questionnaire was methodically structured based on an inductively derived typology of EHEs and TAs from the prior study by Brumec et al. (2023). Respondents reflected on whether they encountered any specified types of EEs during their pilgrimage and experienced described TAs upon returning home. Demographic and religious questions were also included, enabling us to examine correlations between REs, TAs, and these variables.

Through this methodological approach, we aim to delineate the type, frequency, and TAs of REs among pilgrims on the Camino de Santiago, thereby addressing both research questions.

A total of five hundred and one people responded to the online solicitation. Among the respondents, 55% were pilgrims who had traveled the Camino de Santiago once, 21% had completed the pilgrimage twice, and 24% had done so more than twice. The mean age of respondents was 57.5 years (SD = 11.5), with ages ranging from 20 to 80 years. The sample consisted of 41% male and 59% female participants. The respondents were highly educated, with over 79% holding at least a bachelor’s degree.

## Findings and Results

### The Religious Experience of Pilgrims

The cluster delineating the religious experience (Brumec et al., 2023) comprises seven key concepts: (1) connection with a transcendent entity, (2) experiencing bliss, (3) feeling joy, (4) experiencing pleasure, (5) feeling happiness, (6) experiencing gratitude, and (7) feeling grace. This cluster is defined as the experience of a direct connection with God or some other transcendent or supernatural entity. According to data from the survey by Lavrič et al. (2022), 43.1% of respondents reported experiencing RE during the pilgrimage, while 23.8% of respondents were unsure or answered “maybe” regarding their experience of RE, and 33.1% of respondents stated that they did not experience RE during the pilgrimage.

The REs of pilgrims on the Camino de Santiago can be exemplified through three quotations from the analyzed travelogues. The first, a slightly longer quote from the French pilgrim Potdevin, depicts his encounter with a direct connection with God during his pilgrimage, encapsulating all seven codes that constitute the cluster of religious experience:

“Nothing can describe the presence of the Being. He filled the room with his presence. […] there were no shapes, contours, colors, surfaces, or textures. Only energy. His communication—the association I should say—with my being was complete and whole. […] This was the highest possible state of satisfaction and peace in which I was sinking. Joy with a slight sense of awe at such size and grandeur, which could have crushed me if it had not been entirely benevolent. I wished it had never ended: I cannot remember ever having tasted such inner peace and pleasure in my life. I suppose it was that deep and real joy. Nothing in the world can produce such a state. […] In contact with him, I fully experienced love as osmosis: our communication was complete, it took place in thoughts, in actions, in complete feelings, and incomplete understanding, as if, by translating his immediate presence, I had had a joyful encounter […] I was […] in an ecstasy of indescribable happiness […] I was intoxicated by the joys with which He lavished me generously and with boundless extravagance. He showed me the honor and incredible grace […] In a state of bliss, I was filled with immense gratitude and did not know how to thank Him for this grace or how I deserved to be here […] The bliss was complete. At that moment, there was undoubtedly a fundamental thirst in me, a thirst for true happiness, for which every human being rightly longs. When we drink this water, we are no longer thirsty for anything. There is no greater and more perfect happiness […] in the most beautiful bliss. […] A deep source of peace and pleasure” (Potdevin, 2013, pp. 42–43).

Similarly, an Irish Jesuit shares a deeply consoling experience of encountering God’s freely given love, emphasizing a sense of giftedness and grace that felt entirely unearned: “This experience of living in God’s love freely given was deeply consoling and satisfying. I had a strong sense of that “giftedness”, grace, or blessing as I had not earned or deserved this in any way. … it felt like I was born again. I thrashed the salty brine with my fists for pure joy. … Undeniably the Spirit was speaking to me directly” (McManus, 2014, ch. 16/para. 27–29).

American MacLaine’s religious experience took on a unique form, as she described a connection with the angel Ariel:

“I felt a slight shiver up and down my spine, and then a presence seemed to surround me. I recognized the “vibration” of the presence. I even had a name for it. It was an angel, and I felt its name was Ariel. I felt I was being visited by an angel named Ariel, and it began to talk to me in my head. I couldn’t tell if the angel was male or female or both, like a genderless spirit. “Do not be afraid of your physical body,” it said. “Learn to have pleasure as you experience it. Your journey is to learn that. Tune in to the experience and drop your orientation of accomplishing a goal. The goal is the path.” Then the vibration seemed to dissipate, as though the angel had left” (MacLaine, 2000, p. 34).

The connection with a transcendent entity among pilgrims is commonly associated with a state of bliss, joy, pleasure, happiness, gratitude, and grace. These sentiments find expression in the words of pilgrims drawn from the travelogues under analysis.

Potdevin (2013) articulates the dimension of grace, describing his receptivity to what he terms “the fifth dimension of the world, that is, the dimension of grace” (p. 5). Furthermore, he writes about “a state of grace” (p. 88), and “a world of grace” (p. 119), mentioning that “the doors of grace have been opened” (p. 120), and reflecting on how God has shown him “immensely grace” (p. 43) and “incredible grace” (p. 44). He eloquently conveys that God overwhelmed him “with his grace, overwhelmed with great pleasure: he took me over as if I were being bathed in a powerful stream of water, filling me with graces, especially from the inside” (pp. 55–56).

In the last quotation, Potdevin also references the word “pleasure”, which is similarly associated with REs. Another pilgrim, Artnik Knibbe (2020), provides insight into this aspect, stating, “[…] pleasure is not a problem if you are aware of it and do not attach yourself to it. You should allow it to be a part of a free-flowing experience, but not your guide or compensation” (p. 282). She concluded that “the time for pleasure had come” (p. 107).

In addition to grace and pleasure, REs are also closely linked to feelings of gratitude. McManus eloquently describes this dimension as being “lost in gratitude” (ch.13/para.15), feeling that “gratitude overcame me like a warm glow” (ch. 8/para. 15), being “filled with a warm gratitude” (ch. 13/para. 17) and experiencing “the glow of gratitude” (ch. 13/para. 20). In addition to these expressions of gratitude, REs are often intertwined with feelings of bliss, as indicated by MacLaine (2000), who shares, “I had begun to feel blissful” (p. 74) and “I was walking blissfully thinking I was at one with God” (p. 76). Moreover, there are strong associations between REs and feelings of joy and happiness, as articulated by MacLaine (2000), who describes these experiences as “so much joy and happiness” (p. 218). MacLaine further contends that “we as humans have a moral obligation to seek joy. Then we will be in alignment with the Divine” (MacLaine, 2000, p. 10).

Similarly, Jean-Cristophe Rufin (2016), the author of one of the analyzed travelogues, echoes this sentiment by expressing that “joy gave me wings” (ch. 19/para. 3) and that “the feelings of exaltation, joy, and peace get stronger and stronger” (ch. 27/para. 2), emphasizing the “joys of life” (ch. 16/para. 13) that accompany these religious experiences.

### The Transformative Aftereffects of the Religious Experiences

**Fig. 1:**
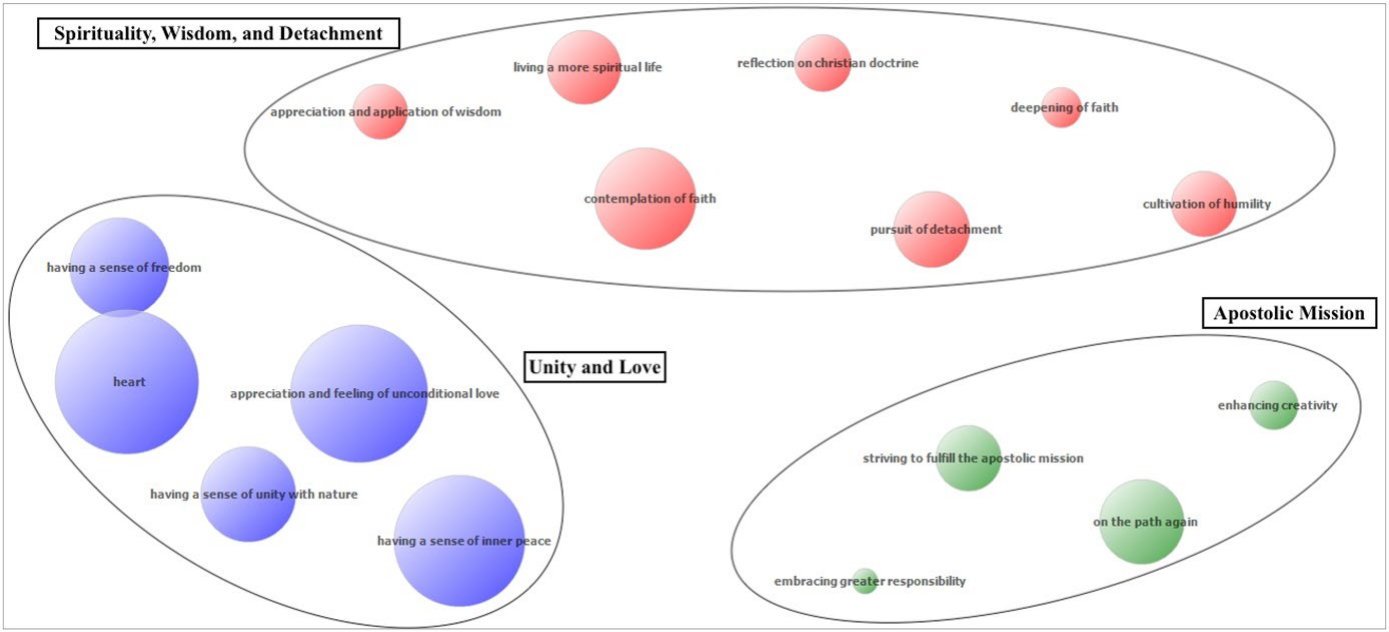
Three types of transformative aftereffects. Note: The size of bubbles indicates how frequently a given code occurred in all the analyzed texts. The proximity of the bubbles represents their average proximity/co-occurrence in the analyzed texts

Religious experience exhibits a robust correlation with three TAs (Table 1). According to the findings of Lavrič et al. (2022), nearly 83% of pilgrims report experiencing Spirituality, Wisdom, and Detachment, over 65% undergo Apostolic Mission and almost 55% embrace Unity and Love. These clusters, depicted in Fig. 1 along with their associated identified codes, represent specific types of TAs of REs that we will analyze in further detail below.

**Table 1:** Top three TAs most strongly correlated with RE and frequencies of pilgrims who experienced these TAs of RE

Exceptional experience	Transformative aftereffect	Pearson’s r	%
Religious experience	Spirituality, Wisdom, and Detachment	0.384**	82.8
Religious experience	Apostolic mission	0.317**	65.2
Religious experience	Unity and Love	0.254**	54.8

**Correlation is significant at the 0.01 level (2-tailed)

*Correlation is significant at the 0.05 level (2-tailed)

### Spirituality, Wisdom, and Detachment

The TAs categorized under Spirituality, Wisdom, and Detachment indicate that many pilgrims experience a shift toward living more spiritual lives upon returning to their everyday routines. They engage in deeper contemplation of human wisdom and faith, resulting in heightened humility and decreased attachment to material possessions, individuals, or relationships. Figure 1 visually depicts the emergence of seven distinct codes within this thematic category, while Table 2 provides reference quotations from analyzed travelogues corresponding to each code.

**Table 2:** Transformative aftereffect Spirituality, Wisdom, and Detachment

Spirituality, Wisdom, and Detachment	
Codes in cluster	Reference quotations from travelogues
*Intellectual dimension*	
Contemplation of faith	“Faith is the most powerful force we know… faith in God, faith in others, faith in ourselves… That’s why we don’t need any organization. Everyone carries faith within themselves, and it is their greatest source of encouragement. Faith is an inner wellspring of strength… it restores health, heals wounds, and, most importantly, banishes fear. Fear is real… the most formidable force we know. But faith is stronger! Faith will always triumph over fear!” (Steblovnik, 2012, p. 205)
	“I have placed all my faith in myself. I’ve placed little in those around me and virtually none in God. More than anything else, I want the faith that guides me, that pushes you along, to be the same faith that guides my children” (Gray & Skeesuck, 2017, pp. 217–218)
	“Faith is not a consequence of action, but a state of being” (Artnik Knibe, 2020, p. 189)
*Ideological dimension*	
Reflection on Christian doctrine	“For hours and hours, I delved into the Song of Songs and Origen’s homilies. A marvelous illumination! I was discovering how much influence this light had on fundamental Christian spirituality” (Potdevin, 2013, p. 99)
	“Somewhat late at night, aware of this gaping void, I opened the Gospel to read the short passages about the Lord’s ascension, hoping to find an answer. I listened with the other ear to the broadcast of the Paris Catholic radio” (Potdevin, 2013, p. 144)
	“I realized that the pinnacle of complete union with God is attainable already in this life. And I thought it good to try to reach it again because it brings a definitive fullness. I found the description of the climax in the High Song, which sings of the love between the soul and its divine Bridegroom. I found that this kind of marriage union has been lived by many Christian mystics for centuries” (Potdevin, 2013, p. 119)
*Public and private practice*	
Living a more spiritual life	“I am beginning to realize that spirituality is a matter of inner shifts, not external ones and that every method works if done correctly. I understand that spirituality is like a sport—it needs practice, proper practice, just like any sport. The knowledge I already had internalizes because I’m doing this with my entire being, not just my head. I begin to deal with my own personality, which, even after the Camino, does not end for me” (Steblovnik, 2010, p. 213)
	“People say they don’t have time to practice some form of spirituality. You practice it in everyday tasks; that’s essential. Practice it when you’re stuck in traffic, practice it when your three kids are at home screaming and making a mess, practice it when …” (Škarja, 2017, pp. 89–90)
	“I know that all the spiritual wisdom I have gained so far cannot help me if I cannot live it in practice. I think that the essence of all life wisdom lies in living them. The theory is always very beautiful, and you can quickly conquer it, but making use of it in everyday life, that is art” (Jernejčič, 2014, p. 85)
*The experiential dimension*	
Deepening of faith	“Of course, I could find that specific instance and, for example, explain that my iPhone stopped recognizing me because I bookmarked pages with information about the hours of adoration and masses throughout Paris for the entire day. That I subscribed to the daily electronic delivery of the word of God and that now I use my iPhone more for praying the breviary—those wonderful moments of prayer with the psalms that give a Christian the rhythm of the day” (Potdevin, 2013, pp. 143–144)
Appreciation and application of wisdom	“Suddenly, deep down, I felt how precious life is. I realized that it is the most valuable thing and that I am already infinitely rich with it. Well, every well-bred child already knows this, you will say, but it’s one thing to talk about it and convince others about it and another to feel it” (Novak, 2004, p. 43)
	“I stopped listening to my soul’s messages with my mind. I stopped trying to capture them in a small bottle. I also did not make any notes on the Way, because I knew. There was no time, space or need for eternalizing or chasing knowledge that was a part of me” (Artnik Knibbe, 2020, p. 170)
	“I felt something I couldn’t define... a kind of knowing” (MacLaine, 2000, p. 289)
Pursuit of detachment	“Paths like these teach me about detachment … If you think you can only live in the house, you’re in, you become obsessively attached to it out of fear of losing it. If you have only one person you can talk to, you become obsessively attached to them out of fear of losing them. And this excessive attachment leads to irrational, sometimes horrifying actions, and thoughts … Because you would do anything to not lose it (whatever it may be). People kill out of fear of losing their land. People lie out of fear of losing a partner. People resort to violence out of fear of losing control. And all of this is just a consequence of excessive attachment” (Škarja, 2017, p. 122)
	“When we are completely unattached to ourselves and without everything when we contemplate the divine, we can finally grow into true joy, into the source of life” (Potdevin, 2013, p. 69)
	“The path has a first and important virtue: it helps to leave home, to step out of oneself, to burn bridges behind, offering complete detachment, impoverishment, and exile. We leave our abode. We leave our dwellings; we leave visible and invisible chains. It sets us in motion. While walking, we acquire a name, a name with feet, worn out and nothing more, just as it should be. Far behind, we leave everything false” (Potdevin, 2013, pp. 90–91)
	“Let me tell you that this is a truly special aspect of the Camino, where you train nonattachment daily. You fully surrender to the flow of meeting people that you will be very fond of or fall in love with a place, but the very next day must leave it. This requires the mastery of detachment while getting the power of flow in return. You don’t cling to any momentary happiness but allow life to bring you more of it. New places, new beauty, new people come our way. It’s a very interesting approach to life. Starting from the perspective that attachments set limits, limits set borders, and those confine our unlimited soul” (Artnik Knibbe, 2020, p. 280)
	“I am grateful that I feel detachment from things, something I wouldn’t have embraced so lightly before the Camino” (Škarja, 2017, p. 186)
Cultivation of humility	“For the first time in my life, I felt the wish to be humble. Until that moment, I had associated humility with subservience, servitude, and spinelessness. Wrong! Humility does not mean bowing before someone, but rather bowing to them. Humility is an acknowledgment of the truth. The humble look upon themselves honestly and objectively. We need not over- or underestimate ourselves, but only recognize our limitations. Only then can we give joy to others and show them that they are just as important as everyone else in the world. In this way, humility bears its fruit: peace of mind, openness, and joy” (Brumec, 2016, p. 87)

Huber and Huber (2012) identified five core dimensions of religiosity, encompassing both sociological and psychological perspectives, which denote distinguishable modes of representing religious content. These dimensions include thought (intellectual and ideological), action (public and private practice), and experience, emotion, and perception (experiential). Analyzing this thematic area through Huber’s framework reveals alignment with these dimensions of religiosity.

Firstly, two codes correspond to thought, reflecting engagement with faith-related concepts. Within the intellectual dimension, there is evidence of contemplation of faith, while in the ideological dimension, there is a thoughtful reflection on Christian doctrine (Platovnjak & Svetelj, 2022). Pilgrims often engage in contemplative practices upon returning home, exploring faith-related concepts, and reflecting on Christian teachings.

Secondly, within Huber’s dimensions of public and private practice, actions are emphasized through “living a more spiritual life” and “deepening of faith.” “Living a more spiritual life” signifies the adoption of profound values into daily existence, integrating spiritual principles into everyday actions. This highlights the importance of incorporating spirituality into daily life. “Deepening of faith” encapsulates private practices within personal religious constructs, involving patterns of action and personal devotion to transcendence.

The experiential dimension is reflected in codes emphasizing emotional and perceptual aspects such as “appreciation and application of wisdom,” “pursuit of detachment,” and “cultivation of humility.”

Wisdom, as encapsulated in the code “appreciation and application of wisdom,” surpasses mere knowledge, delving into a realm of sacred and intuitive insight. It involves discerning the best course of action or way of being for the greater good, as noted by Larry Culliford (2020). This wisdom is not confined to specific beliefs, rather it is grounded in personal and spiritual experiences, fostering a sense of unity with oneself, others, nature, and the divine.

Levenson et al. (2002) view wisdom as a developmental process of self-transcendence, enabling individuals to gain awareness of human nature and break free from societal conditioning. This ability to move beyond self-centered consciousness is vital for fostering empathetic connections with others, as emphasized by Pascual-Leon (1990).

Additionally, according to Sahdra et al. (2010), wisdom plays a crucial role in detachment, characterized by understanding the impermanent and interdependent nature of mental constructs. Religious experiences can facilitate the cultivation of wisdom and detachment, inspiring a desire to detach from worldly concerns and fostering spiritual growth.

Dallh (2018) argues that detachment is the pinnacle of virtue, enabling individuals to align themselves with God and liberate themselves from attachment to worldly matters. Non-attachment, as described by Sahdra et al. (2010), involves releasing rigid ideas and embracing the flow of events, leading to objective perception, compassion, and freedom from afflictive emotions.

Sahdra et al. (2015) define nonattachment as accepting and letting go of life events, possessions, and mental processes, associated with positive traits like mindfulness and self-compassion. Agrawal and Jaiswal (2013) suggest that nonattachment entails accepting life without psychological dependency on outcomes. Siah et al. (2020) propose that nonattachment involves maintaining goals without clinging to outcomes, leading to greater happiness.

The code “cultivation of humility” signifies a shift in the emotional and perceptual experiences of pilgrims, reflecting their newfound understanding of humility. Research by Snow et al. (2023) highlights the transformative role of humility, showing its dynamic relationship with receiving God’s grace and orientation toward oneself, and others, and a reliance on the divine.

### Apostolic Mission

Apostolic Mission stands out as one of the prominent TAs of RE, reflecting pilgrims’ dedication to sharing the insights gained during their journey. This cluster comprises four distinct codes (Table 3), showcasing a sense of duty and responsibility to disseminate their pilgrimage experiences. Furthermore, embracing the Apostolic Mission involves a heightened sense of creativity and responsibility in imparting the teachings of the Camino to others.

**Table 3:** Transformative aftereffect Apostolic Mission

Apostolic mission	
Codes in cluster	Reference quotations from travelogues
*The dimension of action*	
Embracing and striving to fulfill the Apostolic Mission	“Apostles are all of us because each of us, in a way, announces our truth to people around us. Nowadays, we do not call ourselves apostles, but we have different names on the way through history. We are all the same Self, we are all One, we are all connected, so we are all connected with some fragment of ourselves with the vibrations of Jesus and his apostles” (Vranjek, 2015, p. 189)
On the path again	“Amid occasional darkness, a glimmer of hope shines—the Reset key is always available: there exists a space, a world not far from me, where the overwhelmed system can be rebooted. It costs little, it lasts briefly, whether a complete restart or just a screen refresh. With its golden guarantee, this option will accompany me until the end of my days” (Remškar, 2017, p. 74)
*The Experiential dimension*	
Enhancing creativity	“I would prefer to stop writing here. However, the light I discovered in this writing and the role we have as witnesses encourage me to continue. Perhaps some curious reader will still manage to unveil a piece of the opaque veil that conceals lost mystical wisdom and will be seized by the desire to seek even deeper. To embark on the journey and directly encounter the Other” (Potdevin, 2013, p. 77)
	“If the reading before you trigger even the slightest shift within you and within this mysterious field of tightly interconnected energies, our purpose will be fulfilled” (Djura Jelenko & Jelenko, 2010, p. 10)
	“I know that reading this book will at least slightly redirect her current thoughts. Another confirmation that our decision was correct. Share your experiences, and His protection will multiply” (Gričnik, 2018, p. 38)
Embracing greater responsibility	“As I realized at Camino, it’s all up to me. I must take life into my own hands and be my own master. Which means taking responsibility for actions and standing behind one’s decisions” (Jernejčič, 2014, p. 108)
	“No, the Camino is not an ordinary route. It is not innocent, and it is different, perhaps a little wild, and unpredictable. It throws you back into first class but thank goodness for the possibility of retaking the exam. It offers you a chance, nice on a plate. To make you realize, what you’ve ‘known’ for a long time, but don’t know. To forgive yourself and to finally really take on this much-sung, so glorified, so beautifully fucked-up responsibility” (Grešak, 2017, p. 30)

Within Huber and Huber (2012) dimensions of religiosity, two codes underscore actions associated with the Apostolic Mission. Firstly, “embracing and striving to fulfill the Apostolic Mission” emphasizes pilgrims’ proactive engagement in spreading their experiences and insights. Secondly, “on the path again” highlights the phenomenon of repeat pilgrimages among participants of the Camino de Santiago. Survey results from the study conducted by Lavrič et al. (2022) indicate the prevalence of this trend, with 55% of pilgrims completing the route once, 21% walking it twice, and an additional 24% repeating the pilgrimage more than twice.

Psychological research suggests that encountering unusual and unexpected events can foster creativity by evoking mixed emotions and heightening sensitivity to novel associations and ideas. The findings of Brumec’s (2024a) survey underscore the significant impact of the Camino de Santiago pilgrimage on enhancing creativity. Similarly, the present study suggests that this pilgrimage experience can serve as a catalyst for fostering creativity, as evidenced by the code “enhancing creativity”. Many travel narratives about the Camino de Santiago can be viewed as the realization of this theme, often serving as the debut work for authors. Three quotations reflecting this theme from such inaugural works are illustrated in Table 3.

The fourth code in this cluster, “embracing greater responsibility,” indicates that pilgrims frequently assume more significant responsibilities following the completion of their pilgrimage. This reflects a deeper commitment to their spiritual journey and a heightened sense of duty toward others.

These final two codes, “enhancing creativity” and “embracing greater responsibility,” align with one of Huber and Huber (2012) dimensions of religiosity, specifically the experiential dimension.

### Unity and Love

The TA concerning Unity and Love emerges as a significant aspect of pilgrims’ experiences upon returning home, encompassing five distinct codes (Fig. 1). These codes reflect pilgrims’ emotional shifts toward prioritizing love, unity with nature, and following their hearts, as expressed in references from travelogues provided in Table 4. This thematic area aligns with Erich Fromm’s (2006) perspective on brotherly love, emphasizing unity, solidarity, and oneness inherent in this form of love. Fromm suggests that brotherly love transcends differences, recognizing interconnectedness and shared humanity.

**Table 4:** Transformative aftereffect Apostolic Mission

Unity and Love	
Codes in cluster	Reference quotations from travelogues
*The experiential dimension*	
1. Appreciation and feeling of unconditional love	“Undeniably, we are the front lines of God’s provision to the world. If we believe Jesus left his Holy Spirit among us, then we must embrace the fact that He charges us with the very task of loving the world. Loving the world on his terms. Loving the world unconditionally and passionately meeting the needs of others, of those who are broken. We are his hands and feet. We are his provision for the world” (Gray & Skeesuck, 2017, p. 231)
2. Having a sense of inner peace	“We are the vessels through which the world can know God’s love. In this kind of love, we find the ability to be honest about our fears and the freedom to share our failings. We find a different kind of bread to relieve a seemingly insatiable hunger, and a different kind of wine to satisfy a seemingly unquenchable thirst. Just as our bodies hunger for food and drink, our hearts long for love, our souls long to be pursued” (Gray & Skeesuck, 2017, pp. 178–179)
3. Having a sense of unity with nature	“Respecting and loving nature, animals, other people, and oneself is the essence of our life. We should ask ourselves whom we love and who we don’t, who loves us. We should put more effort into unconditional love and love someone just because they exist” (Jernejčič, 2014, pp. 49–50)
4. Having a sense of freedom	“Camino is an understanding that the essence of life is love” (Škarja, 2017, p. 87)
	“I know that reading this book will at least slightly redirect her current thoughts. Another confirmation that our decision was correct. Share your experiences, and His protection will multiply” (Gričnik, 2018, p. 38)
	“In silence, I became aware of the sanctitude of life and the space that I am part of. With sincere modesty arising from the awareness of magnitude, I surrender to life or, better put, to love. Life, in fact, is pure love” (Artnik Knibbe, 2020, p. 260)
	“Although my daily rhythm has caught me once again in its net, it often helps me when I re-experience the infinite freedom and the magical power of the human body and spirit and their connection with nature and other people” (Djura Jelenko & Jelenko, 2010, p. 140)
5. Heart	“Silence is necessary to feel peace in the heart. When we are in it, we are united with all of nature, with everything that surrounds us. This is love” (Jenko Simunič & Jenko, 2014, p. 67)

The codes within this cluster resonate with Huber and Huber (2012) experiential dimensions of religiosity, encompassing aspects of experience, perception, and emotions. Shedding light on the transformational effects of Unity and Love, particularly evident in codes such as “heart” and “appreciation and feeling of unconditional love,” is McGilchrist’s (2018) perspective, notably his metaphor of the “heart brain” and the role of the brain’s right hemisphere. McGilchrist explains the intricate connections between the right hemisphere, neurons in the skin and around the heart, and the body’s surroundings, allowing for the metaphorical concept of the “heart brain.”

The rise of capitalism marked a shift from altruism to efficiency and competition, leading to a significant conflict between self-interest and universal brotherliness. In late modernity, competition has become dominant, driven by individuals striving for social status and recognition. Gergen (2009) highlights competition as a manifestation of individualistic tendencies, prioritizing achievement and perpetuating inauthentic relationships. Addressing this challenge involves prioritizing emotions to foster genuine connections, beyond exclusive rational thinking. The Camino de Santiago pilgrimage offers such an opportunity, as many pilgrims develop the ability to quiet their minds and follow their hearts, as illustrated in Petra Škarja’s travelogue (2017): “And what’s in it for you? The mind might say, but here on the Camino, it is easy to silence it and follow the heart” (p. 88).

#### Correlation Between Religious Experiences, Transformative Aftereffects, and Demographic/Religious Variables

The correlation analysis between REs, TAs, and demographic/religious variables is presented in Table 5.

**Table 5:** Correlation coefficients between RE, TAs, and demographic/religious variables

RE and TAs	FE	AG	ED	IG	RP
Religious experience	− 0,005	0,048	0,128**	0,469**	0,536**
Unity and Love	0,046	− 0,088	− 0,082	0,099*	0,074
Spirituality wisdom and detachment	0,081	− 0,084	− 0,037	0,171**	0,178**
Apostolic mission	− 0,097*	− 0,035	− 0,017	0,154**	0,188**

Note: *GE* gender, *AG* age, *ED* education, *IG* importance of God, *RP* frequency of religious practice; for gender was used the Kendall’s tau-b coefficient; for age, education, the importance of God, and frequency of religious practice were used Pearson’s correlation coefficients

**Correlation is significant at the 0.01 level (2-tailed)

*Correlation is significant at the 0.0 level (2-tailed)

The analysis revealed a positive correlation between the importance of God and engagement in religious practices, underscoring the interconnectedness between one’s emphasis on spirituality and active involvement in religious activities. This suggests a heightened likelihood of experiencing REs during pilgrimages.

Our study also revealed a positive correlation between educational attainment and the occurrence of REs. As detailed in the ‘Distribution of Pilgrims Reporting Religious Experiences by Level of Formal Education’ (Table 6), nearly 85% of pilgrims who reported REs held at least a bachelor’s degree, with 40.5% having a bachelor’s degree and 44.3% possessing a master’s degree or higher.

**Table 6:** Distribution of pilgrims reporting religious experiences by level of formal education

Highest level of formal education	No		Maybe		Yes		Total	
Elementary school or less	1	50,0%	0	0%	1	50,0%	2	100,0%
High school	38	38,0%	31	31,0%	31	31,0%	100	100,0%
Bachelors degree	70	35,0%	45	22,5%	85	42,5%	200	100,0%
Masters degree or more	51	27,7%	40	21,7%	93	50,5%	184	100,0%
Total	160	32,9%	116	23,9%	210	43,2%	486	100,0%

The common trend indicating lower religiosity among highly educated individuals (Sacerdote & Glaeser, 2001; Voas & McAndrew, 2012) was recently confirmed by Zinkina et al. (2021), who analyzed data from seven waves of the World Values Survey. Given this trend, it may seem surprising that our study found a positive correlation between educational attainment and the occurrence of REs. However, this finding does not necessarily imply greater religiosity among highly educated individuals. Instead, it suggests that those with higher levels of education may be more inclined to report REs during their pilgrimages. This result indicates that the relationship between education and the inclination to report religious experiences, as well as religiosity, may be more complex and context-dependent than previously understood. It underscores the need for further research to explore these dynamics in greater depth.

Moreover, individuals who prioritize God and engage in religious practices tend to experience elevated levels of Spirituality, Wisdom, and Detachment (Platovnjak & Svetelj, 2024). This prioritization is also associated with a stronger sense of Apostolic Mission, reflecting a heightened purpose and commitment to sharing spiritual insights. While there is a slight tendency for females to report lower levels of Apostolic Mission, those prioritizing God tend to report significantly higher levels of Unity and Love. These findings highlight the intricate interplay between religious beliefs, practices, and transformative experiences during pilgrimages.

## Limitations

This study has several limitations. First, we assume that pilgrims’ travelogues provide credible insights into their Camino de Santiago experiences. While these travelogues offer authentic personal narratives, they are subjective and may not fully reflect objective reality. Given our study’s ontological realism and epistemological constructivism, we consider these narratives a valid basis for our conclusions. We also aimed to minimize bias during data analysis.

Second, travelogues were intended for a general audience, which may lead authors to alter or embellish their experiences. While this could impact the transferability of our findings, we believe it does not significantly affect our core conclusions.

Third, our findings are based exclusively on Slovene-language travelogues, which may not fully represent the experiences of pilgrims from other linguistic or cultural backgrounds. Additionally, the survey was conducted online, limiting participation to those with internet access and potentially skewing results toward members of Facebook groups, who may have been more likely to report positive experiences.

## Conclusion and Implications

These findings underscore the transformative impact of REs during pilgrimages, leading to heightened Spirituality, Wisdom, Detachment, and a sense of mission to share insights, alongside an increased focus on love and unity. These enduring transformations significantly contribute to personal growth and life perspectives among pilgrims.

Highlighting significant correlations, this study emphasizes the importance of prioritizing one’s relationship with God and engaging in regular religious practices, consistently associated with elevated spiritual growth, wisdom, detachment, and a reinforced sense of Apostolic Mission.

From a sociological perspective, these implications suggest that religious and spiritual factors play a pivotal role in shaping the TAs of REs. Prioritizing spirituality fosters greater spiritual growth, wisdom, a sense of mission, and active involvement in sharing faith or spiritual insights, informing discussions on faith, spirituality, and personal transformation.

Contributing to a deeper understanding of REs among Camino de Santiago pilgrims, this study sheds light on the fostering of Unity and Love, which may promote tolerance, empathy, and compassion in society. Additionally, the emergence of an Apostolic Mission suggests that pilgrims may engage in altruistic or community-oriented activities, positively benefiting their communities.

Encouraging further research into REs and their TAs, both on the Camino de Santiago and in other pilgrimage contexts, can deepen our understanding of the spiritual and psychological dimensions of such journeys. Given the emphasis on Unity and Love, these findings may also facilitate interfaith dialogue and foster better interpersonal relations across religious and cultural boundaries.

## Appendix

See Table7

**Table 7:** List of analyzed travelogues

Author	Published	Walked	Title
1. Artnik Knibbe, Tjaši	Artnik Knibbe (2020)	2014	Vulnerable: My journey to oneness on the Camino de Santiago
2. Božič, Saša	Božič (2018)	2018	Storyteller: 365 inspirations of the citizens of the world and a story about the path to self
3. Brumec, Snežana	Brumec (2016)	2016	The Camino
4. Djura Jelenko, Saša	Djura Jelenko and Jelenko (2010)	2009	The Camino: A 800 km long experience
5. Jelenko, Vinko	Djura Jelenko and Jelenko (2010)	2009	The Camino: A 800 km long experience
6. Gliha, Franc	Gliha (2018)	2017	The Camino de Santiago “In two parts” 2017–2018
7. Gray, Patrick	Gray and, Skeesuck (2017)	2014	I’ll Push You: A Journey of 500 Miles, Two Best Friends, and One Wheelchair
8. Skeesuck, Justin	Gray and Skeesuck (2017)	2014	I’ll Push You: A Journey of 500 Miles, Two Best Friends, and One Wheelchair
9. Gričnik, Ivan	Gričnik (2014)	2014	My Camino
10. Gričnik, Ivan	Gričnik (2018)	2014	From Santiago to Assisi
11. Jenko Simunič, N. & Jenko, B	Jenko Simunič and Jenko (2014)	2013	Our path
12. Jernejčič, Nataša	Jernejčič (2014)	2012	My path: The Camino de Santiago
13. Kapetanović, Ivan	Kapetanović (2017)	2016	Camino de Santiago: The Way of St. James, Lečevica—Santiago de Compostela
14. Klug, Mojca	Klug (2018)	2015	Camino Francés: My attempt to escape the “nonsense” of modern society
15. Kvaternik, Stanislav	Kvaternik (2015)	2011	My pilgrimage: Along the path of St. James in Santiago de Compostela
16. Lepej Bašelj, Saša	Lepej Bašelj (2009)	2006	Camino: My lonely path or cleansing of soul and body
17. MacLaine, Shirley	MacLaine (2000)	1994	The Camino: A Journey of the Spirit
18. McManus, Brendan	McManus (2014)	2011	Redemption Road: Grieving on the Camino
19. Močnik, Uroš	Močnik (2009)	2007	In 14 days to the end of the world: a journey into inner peace, The Camino
20. Novak, Nace	Novak (2004)	2002	The Camino: From Nova Gorica to Compostela
21. Potdevin, Jean-Marc	Potdevin (2013)	2008	Reset: The mystical experience of a businessman on the footpath to Compostela
22. Remškar, Eva	Remškar (2017)	2017	And here we go: moms and daughters at the Camino
23. Grešak, Mojca	Grešak (2017)	2017	And here we go: moms and daughters at the Camino
24. Rigler, M. & Rigler, M	Rigler and Rigler, (2004)	1999	You are blessed, poor man: A pilgrimage along the path of St. James to Compostela
25. Rufin, Jean-Cristophe	Rufin (2016)	no data	The Santiago Pilgrimage: Walking the Immortal Way
26. Sluga, Rado	Sluga (2017)	2011	In the embrace of the trail: El Camino
27. Steblovnik, Mirjana	Steblovnik 2010	2008	Buen Camino, Peregrino
28. Steblovnik, Mirjana	Steblovnik 2012	2010	All just because of one church at the end of the world: The Camino Portugal
29. Škarja, Petra	Škarja (2017)	2016	The Camino: From slavery to freedom
30. Štuhec, Jožef	Štuhec (2011)	2008	Across the Pyrenees to the Atlantic: A little different Camino
31. Udovič, Vladimir	Udovič (2012)	2010	I am alive, I walk, and I am happy: a travelogue of a pilgrimage to the Camino
32. Vranjek, Bojana	Vranjek (2015)	2014	The Camino: The mysticism of the invisible world

## Footnotes

1 To obtain the Compostela you must: make the pilgrimage for religious or spiritual reasons, or at least with a searching attitude; do the last 100 km on foot or horseback or the last 200 km by bicycle; and collect the stamps on the Credencial del Peregrino from the places you pass through to certify that you have been there (The Compostela). For those undertaking the pilgrimage by sailboat, you must sail 100 nautical miles and complete the rest of the Camino on foot from the port of disembarkation. Pilgrims in wheelchairs should contact the International Pilgrim Welcome Center for specific requirements. Pilgrims Welcome Office, n.d.: https://oficinadelperegrino.com/peregrinacion/la-compostela/ [Accessed 26 August 2024].

2 Between 2002 and 2013, there were 17 articles and book chapters published on the topic of the Camino de Santiago. However, in the following decade (2014–2023), this number increased significantly to 157 publications, indicating a notable surge in scholarly interest in the subject. https://www-webofscience-com.ezproxy.lib.ukm.si/wos/woscc/summary/3df9626b-e31c-4344-b672-ceeed642024d-d5aa4f3b/relevance/1 [Accessed 17 March 2024].

3 Our study did not require ethical approval as it analyzes datasets obtained from published travelogues and online surveys, in which the data are anonymized (we used trusted software that does not allow linking between identifiers and responses).

4 See Appendix for the complete list.
